# Characterization of a mantle cell lymphoma cell line resistant to the Chk1 inhibitor PF-00477736

**DOI:** 10.18632/oncotarget.5954

**Published:** 2015-10-02

**Authors:** Valentina Restelli, Rosaria Chilà, Monica Lupi, Andrea Rinaldi, Ivo Kwee, Francesco Bertoni, Giovanna Damia, Laura Carrassa

**Affiliations:** ^1^ Laboratory of Molecular Pharmacology and Laboratory of Cancer Pharmacology, Department of Oncology, IRCCS- Istituto di Ricerche Farmacologiche “Mario Negri”, Milan, Italy; ^2^ Lymphoma and Genomics Research Program, IOR Institute of Oncology Research, Bellinzona, Switzerland; ^3^ Dalle Molle Institute for Artificial Intelligence (IDSIA), Manno, Switzerland; ^4^ Swiss Institute of Bioinformatics (SIB), Lausanne, Switzerland; ^5^ Lymphoma Unit, IOSI Oncology Institute of Southern Switzerland, Bellinzona, Switzerland

**Keywords:** Chk1, cyclin D1, mantle cell lymphoma, targeted therapy, mechanisms of resistance

## Abstract

Mantle cell lymphoma (MCL) is an aggressive B-cell lymphoma characterized by the chromosomal translocation t(11;14) that leads to constitutive expression of cyclin D1, a master regulator of the G1-S phase. Chk1 inhibitors have been recently shown to be strongly effective as single agents in MCL. To investigate molecular mechanisms at the basis of Chk1 inhibitor activity, a MCL cell line resistant to the Chk1 inhibitor PF-00477736 (JEKO-1 R) was obtained and characterized. The JEKO-1 R cell line was cross resistant to another Chk1 inhibitor (AZD-7762) and to the Wee1 inhibitor MK-1775. It displayed a shorter doubling time than parental cell line, likely due to a faster S phase. Cyclin D1 expression levels were decreased in resistant cell line and its re-overexpression partially re-established PF-00477736 sensitivity. Gene expression profiling showed an enrichment in gene sets involved in pro-survival pathways in JEKO-1 R. Dasatinib treatment partly restored PF-00477736 sensitivity in resistant cells suggesting that the pharmacological interference of pro-survival pathways can overcome the resistance to Chk1 inhibitors. These data further corroborate the involvement of the t(11;14) in cellular sensitivity to Chk1 inhibitors, fostering the clinical testing of Chk1 inhibitors as single agents in MCL.

## INTRODUCTION

Mantle Cell Lymphoma is a subtype of non Hodgkin Lymphoma characterized by an unfavourable clinical course with a median overall survival of only 4-5 years [1-3]. Although this lymphoma presents an initial high response to first line treatment, its clinical course is characterized by continuous relapses, resulting in a discouraging long term outcome, thus MCL is considered non curable with current therapies and the identification of new and effective therapeutic strategies is of fundamental importance [4]. Deregulation of cell cycle is the characteristic pathogenic hallmark of MCL. Overexpression of cyclin D1 occurs as a result of the chromosomal translocation t(11;14)q(13;32), which fuses the enhancer promoter of the immunoglobulin heavy chain gene with the transcription unit of the CCND1 proto-oncogene, and characterizes all MCL cases. Additional genetic alterations occur in subsets of MCL and are related to the cell cycle machinery and to the cellular response to DNA damage [1, 4]. Specifically, many genetic lesions involve other important components of the G1/S phase cell cycle regulatory checkpoint, such as p16INK4 (deletion), and CDK4 (amplification) which together with the overexpression of cyclin D1 contribute to impair the control of G1-S transition in MCL [5].

Cell cycle checkpoints are constantly activated in the cells after exogenous and endogenous DNA damage to avoid their cycling without having fixed the DNA damage [6]. The serine/threonine kinase Chk1 is a crucial player of the cell cycle checkpoints machinery, being activated after exogenous DNA damage, such as the damage caused by cytotoxic agents, and activating the S and G2 checkpoints, by coordinating various aspects of DNA repair, such as the homologous recombination repair and by inducing apoptosis whenever necessary [7, 8]. In the last years Chk1 inhibitors have been developed in combination with cytotoxic chemotherapeutics (especially antimetabolites such as gemcitabine, hydroxyurea, and cytarabine) to potentiate their genotoxic effects [9-11]. Recently, a role of Chk1 in the absence of exogenous DNA damage for cell proliferation and survival, mostly due to its crucial role in regulating CDKs activity and in controlling DNA replication, has been described and corroborated by many experimental evidence, suggesting that Chk1 inhibitors could also be effective as single agents in tumors with a specific genetic background [12-14]. For example Chk1 inhibition caused cell death in the U2OS osteosarcoma cell line [15], it has been shown to be a crucial therapeutic target in neuroblastoma [16] and in melanoma cell lines with high levels of endogenous replicative stress [17]. Tumor cells deficient in Fanconi anemia pathway are hypersensitive to Chk1 inhibition [18] and Chk1 inhibitors are effective against Myc-driven malignancies, such as certain B-cell lymphomas and some breast and lung cancers [19, 20]. Recently, the protein kinase Wee1 has been shown to be in synthetic lethality with Chk1. Combined treatment with Chk1 and Wee1 inhibitors showed a strong synergistic cytotoxic effect in various human cancer cell lines suggesting that this combined target specific strategy could be a valid therapeutic approach against cancer [21-23]. We recently found that the have activity of the combination of Chk1 and Wee1 inhibitors is strikingly effective in preclinical models of MCL, at extremely low doses, providing a strong rational for its possible successful application in the clinical setting for this disease [24]. Interestingly, MCL cell lines were also extremely sensitive to the Chk1 inhibitor PF-00477736 as single agent. To investigate the molecular mechanisms at the basis of the Chk1 inhibitor activity in MCL, a MCL cell line, JEKO-1, resistant to a Chk1 inhibitor was isolated and characterized.

## RESULTS

### Characterization of JEKO-1 MCL cell line resistant to the Chk1 inhibitor PF-00477736

**Figure 1:**
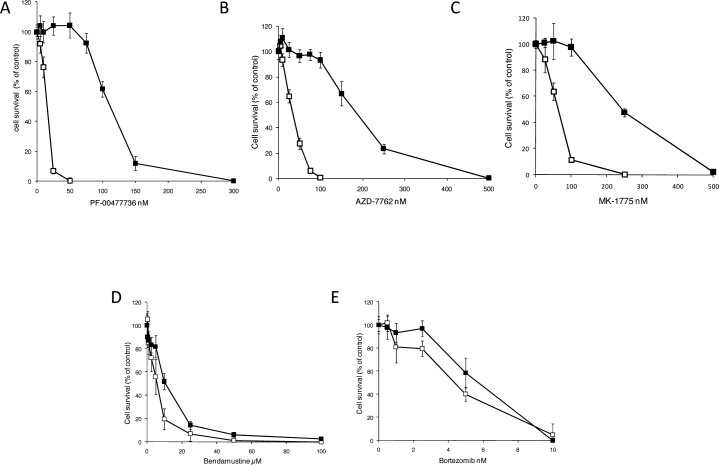
Pharmacological activity of JEKO-1 cell line resistant to PF-00477736 Cytotoxic effect of PF-00477736 (**A)**, AZD-7762 (**B)**, MK-1776 (**C)**, Bendamustine (**D)** and Bortezomib (**E)** in JEKO-1 parental (□) and in JEKO-1 R (■). Data are represented as mean ± SD of three independent experiments.

In order to get more insights on the response mechanisms to Chk1 inhibitors of MCL, we selected a MCL cell line, JEKO-1, resistant to the Chk1 inhibitor PF-00477736 by treating cells for about one year with growing concentrations of PF-00477736 (see details in materials and methods). Figure 1A shows the PF-00477736 sensitivity of the parental (JEKO-1) and resistant (JEKO-1 R) cells. The JEKO-1 R cell line was at least seven times more resistant to PF-00477736 than its parental cell line (IC 50 value of 140±5 nM *vs* 20.6 ±4 nM); the resistance was stable for at least 5 months after isolation and propagation in culture conditions with no drug (experimental conditions used for the subsequent experiments). JEKO-1 R cell line resulted more resistant also to another Chk1 inhibitor (AZD-7762) (IC 50 of 222.6 ±3 nM *vs* 36.7± 2 nM) (Figure 1B). To exclude that the acquired resistance to Chk1 inhibition could be due to higher extrusion of the drug from the cells, MDR-1 (multidrug resistant gene, coding for the ABCB1 ATP-dependent drug efflux membrane pump), MRP-1 (coding for the ABCC1 membrane pump) and BCRP (coding for ABCG2 membrane pump) expression levels were monitored and resulted similarly expressed in the parental and resistant cell lines (Supplementary Figure 1). Moreover, treatment with Doxorubicin, substrate of the three membrane pumps, showed similar activity in the parental and resistant JEKO-1 cell lines (Supplementary Figure 1). Considering the functional inter-relationship and the pharmacological synergism observed treating with Chk1 and Wee1 inhibitors [21], we next evaluated the cytotoxic response of both cell lines to the Wee1 inhibitor MK-1775, and found that the JEKO-1-R cell line was more resistant to this drug as compared to the parental cell line (IC50 of 241±15 nM *vs* 56.8 ± 6 nM) (Figure 1C). On the contrary, sensitivity of the two cell lines to bendamustine and bortezomib, drugs commonly used for the treatment of MCL [25], resulted comparable (Figure 1D-1E). The activity of other DNA damaging agents, that notably activate Chk1, was also evaluated and found to be alike (Supplementary Table 1).

We evaluated the activation of apoptosis in JEKO-1 parental and resistant cell line after treatment for 24 and 72 hours with PF-00477736 at equimolar (15 nM) and at equitoxic IC50s concentrations (15 nM and 150 nM respectively for JEKO-1 and in JEKO-1 R). A caspase 3 activity was detected in JEKO-1 parental at 15 nM, but not in JEKO-1 R at this concentration; however apoptosis could be detected in JEKO-1R cells after treatment with a dose of 150 nM (Supplementary Figure 2A). These data were corroborated by the TUNEL assay performed in the same experimental conditions (Supplementary Figure 2B). Similarly, at the corresponding IC50s in both cell lines, treatment with PF-00477736 induces γH2AX (Supplementary Figure 2C), which persisted longer in JEKO-1R. All these data suggest that resistant cell line still sensed the DNA damage and was able to respond by activating apoptosis.

### JEKO-1 MCL cell line resistant to Chk1 inhibitor PF-00477736 shows a shorter cell cycle and a quicker S phase

**Figure 2:**
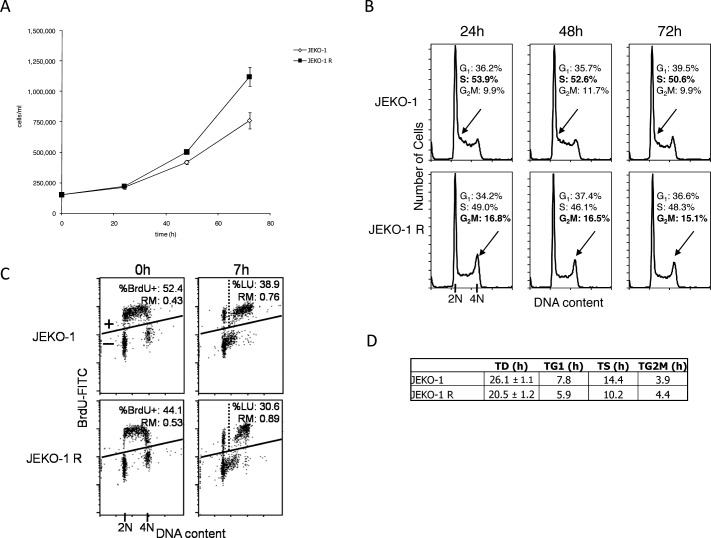
Analysis of cell cycle distribution **A.** Cell growth curves of JEKO-1 parental (◊) and JEKO-1 R (■). Data are represented as mean ± SD of two independent experiments. **B.** Flow cytometric analysis of DNA content at 24, 48 and 72 hrs after seeding. Percentage of cell cycle phases (G1-S-G2/M) are included in the figure. As cells were in exponential growth, DNA distribution remained almost constant over time. The arrow points early S phase in JEKO-1 parental and the more evident G2-M peak in the JEKO-1 R cell line. **C.** Pulse-chase DNA-BrdUrd analysis at the end of 20 min of BrdUrd incubation (0 hr) and 7 hr after BrdUrd washout. Cells were considered BrdUrd-positive (BrdUrd+) when detected above the line. The vertical dashed line separates undivided (right) from divided (left) BrdUrd+ cells. The percentage of labeled undivided cells (%LU) and the relative movement (RM) are indicated in the plots. %LU represents those cells that were in S phase at 0 hr and that are still undivided after 7 hr, while RM represents their average state of completion of DNA duplication. **D.** Table summarizing the doubling time and duration of the different cell cycle phases in JEKO-1 parental and resistant cell lines.

We next evaluated, if any, differences in cell growth of the JEKO-1 R as compared to the parental cell line. Figure 2A shows the cell growth curves of the two cells population; doubling time calculation evidenced a significant difference (*p* = 0.0047) of 6 hours in JEKO-1 R (20.5 hours) versus parental cell line (26.1 hours). FACS analysis was then performed at different time points after cells seeding (Figure 2B). Cell cycle distribution appeared slightly different between the two cell lines with higher percentage of cells in S phase in parental and a more emphasized G2-M peak in the resistant cell line. To better investigate the duration of S phase, BrdUrd pulse-chase analysis was performed in parental and resistant cells harvesting the samples immediately after BrdUrd labeling and after 7 hours; this time point was chosen as previous experiments indicated that it is a time point sufficient to follow cell progression through S phase. This analysis confirmed the higher percentage of S-phase cells in JEKO-1 parental cells than the JEKO-1 resistant ones (52.4 *vs* 44.1 at time 0 and 38.9 *vs* 30.6 at time 7). The higher percentage of S phase cells can be ascribed to a lower DNA synthesis rate and thus to a longer duration of the phase, confirmed by the higher percentage of labelled undivided cells and by lower relative movement (RM) observed at 7 hr in JEKO-1 respect to JEKO-1 R (Figure 2C). The extrapolated duration of the different cell cycle phases (Figure 2D) showed that JEKO-1 R cells display a quicker S phase as compared to the parental cell line and this finding may explain the difference in doubling times observed between the two cell lines.

**Figure 3:**
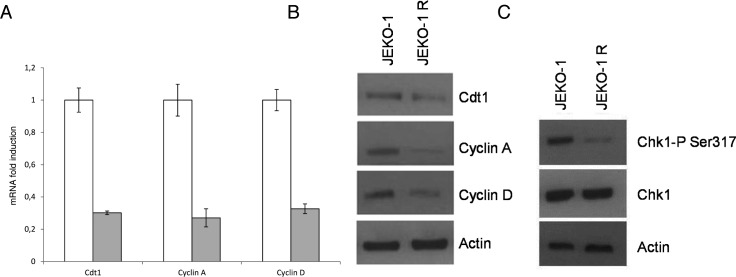
Expression of cell cycle markers **A.** Real time PCR showing cyclinA, Cdt1 and cyclin D1 expression levels in parental JEKO-1 cells (white bar) and in JEKO-1 R cells (grey bar). Data are normalized to the internal mRNA levels of actin and are represented as the fold change from JEKO-1 parental samples. Mean ±SD of three independent experiments. **B.** Western Blot Analysis showing cyclin A, Cdt1 and cyclin D1 and actin protein levels in the parental and resistant cell line. **C.** Western Blot Analysis showing pS317Chk1, Chk1 and actin protein levels in the JEKO-1 and JEKO-1 R cell lines.

We next evaluated the expression of the S phase molecular markers Cdt1 and cyclin A, finding out that they were decreased both at the mRNA and at the protein levels in the JEKO-1 R cell line (Figure 3A-3B). Moreover, based on our previous evidence of an involvement of the translocation t(11;14) in PF-00477736 sensitivity [24], we evaluated the expression of cyclin D1, that the translocation in MCL renders constitutively active, and a decrease in its expression levels (both mRNA and protein) could be observed in JEKO-1 R cell line (Figure 3A-3B and Supplementary Figure 3). A PCR analysis confirmed the presence of the t(11;14) in the resistant cell line (data not shown), suggesting that resistance is not due to the selection of cell clone loosing the t(11,14). In addition, treatments with both 5′aza-deocycitidine and actinomycin D led us to exclude any role of epigenetic and transcriptional regulatory mechanisms in determining lower cyclin D1 levels in JEKO1-R cells (data not shown). We also evaluated Chk1 protein levels and its activating status (phosphorylation in S317) finding out that, comparable total protein levels of Chk1 were associated with a substantial decrease in constitutive activation of Chk1 in the JEKO-1 R cells as compared to the parental cell line (Figure 3C).

### Cyclin D1 expression levels inversely correlate with the PF-00477736 resistance

**Figure 4:**
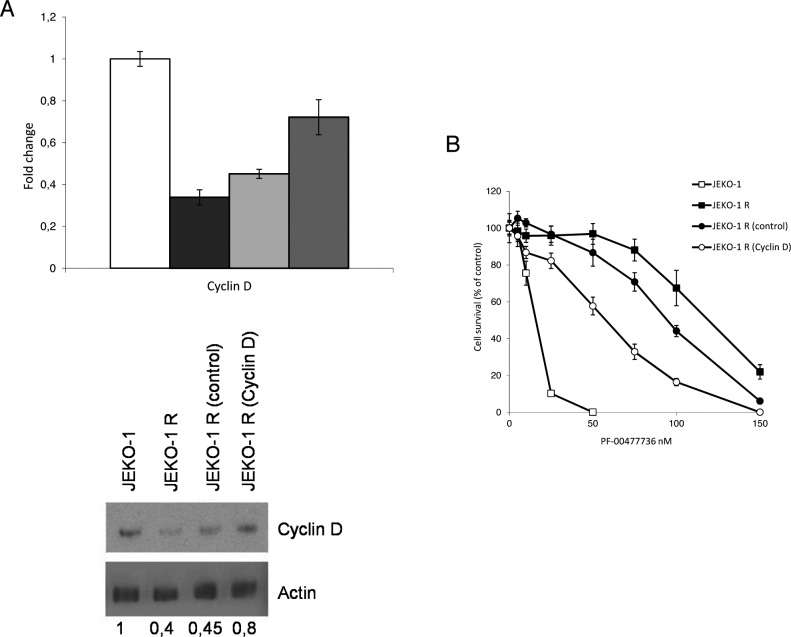
Modulation of Cyclin D1 expression in the resistant JEKO-1 cell line **A.** Real time PCR (upper panel) and western Blot Analysis (lower panel) of cyclin D1 levels in parental JEKO-1 (white bar), JEKO-1 R non infected (black bar) and infected with either control (light grey bar) or cyclin D1 lentiviral vector (grey bar). **B.** Cytotoxic effect of PF-00477736 in parental JEKO-1, JEKO-1 R not infected and infected with either control or cyclinD expressing lentiviral vector. Data are represented as mean ± SD of two independent experiments.

Our previous data suggested that the high activity of the complex CDK4/6-cyclin D1 due to the presence of the t(11;14) could potentially explain the sensitivity to PF-00477736 [24]. To assess if the decrease in expression level of cyclin D1 could be related to the acquired PF-00477736 resistance, we induced re over-expression of this protein in the resistant cell line through a lentiviral expression system. A significant re-overexpression of cyclin D1 as compared to the JEKO-1 R cell line infected with the control empty vector, could be obtained in cyclin D1 infected cells (Figure 4A), although it did not reach the expression levels of the parental cell line. However, when we evaluated the effects of cyclin D1 re-expression on the cytotoxicity to PF-00477736, a partial restoration of the sensitivity to PF-00477736 in the resistant cell line could be observed (Figure 4B).

### Contribution of SRC pathway to the resistance to Chk1 inhibitor

**Figure 5:**
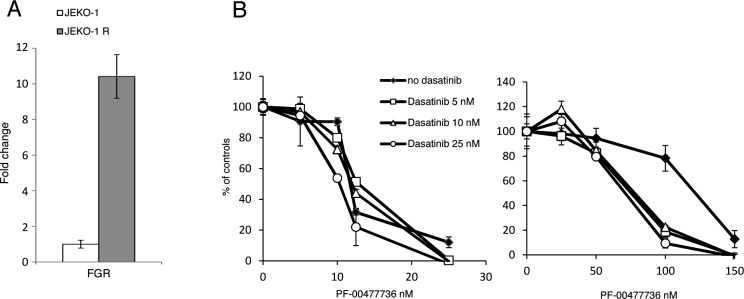
SRC pathway in the JEKO-1 R cell line **A.** Real Time PCR of FGR levels in JEKO-1 parental and resistant cells. Data are represented as mean ± SD. **B.** Cytotoxic effect of PF-00477736 in parental (left panel) and resistant (right panel) JEKO-1 cells either alone or with not toxic concentration of Dasatinib. Data are represented as mean ± SD.

To get further insights in the molecular mechanisms at the basis of the acquired resistance to PF-00477736, gene expression profiles were obtained in untreated parental and resistant cells. The transcripts down-regulated in the JEKO-1 R cell line were enriched of genes involved in cell cycle progression and E2F1 targets (Supplementary Table 2, Supplementary Figure 4), while the up-regulated genes were enriched of gene sets involved in cell survival and proliferative pathways (such as NFKB, SRC/MAPK pathways) (Supplementary Table 2, Supplementary Figure 5). Since *FGR*, coding for a member of the SRC family, was the most up-regulated transcript in the JEKO-1 R cell line (Supplementary Table 3), as also validated by real time PCR (Figure 5A), we decided to test the combination of PF-00477736 with the clinically available dual Src/Abl kinase inhibitor dasatinib [27]. A partial restoration of PF-00477736 sensitivity was observed in the resistant cell lines when the Chk1 inhibitor was combined with non-toxic concentrations of dasatinib, although the latter, as single agent, did not show differences of activity between the parental and the resistant JEKO-1 cells (Figure 5B and Supplementary Figure 6A). When we investigated the cytotoxicity to PF-00477736 in the presence of dasatinib in the MCL cell line REC-1 with a primary resistance to Chk1 inhibitors [24], no reversion of drug resistance could be observed, suggesting that in this experimental system other molecular mechanisms are taking place (Supplementary Figure 6B).

## DISCUSSION

Chk1 is a key regulator of the S and G2 checkpoints, replication initiation and replication fork stability, being required during normal S phase to avoid deleterious DNA breakage and to ensure maintenance of genome integrity [7, 12]. In recent years the development of Chk1 inhibitors has been considered to enhance the effectiveness of chemotherapeutic drugs [28]. However, the emerging evidence of the crucial role of Chk1 during normal cell cycle progression, prompted the study of the effect of Chk1 inhibitors as single agents, as an alternative therapeutic strategy for the treatment of some tumors. The identification of the molecular markers responsible for the extreme sensitivity to Chk1 inhibitors of some tumors is of paramount importance because it could provide tailoring opportunities to stratify the most responsive patients maximizing the effects of this targeted therapy. We have recently demonstrated that MCL cells are extremely sensitive to the Chk1 inhibitor PF-004777736 as single agent, much more than other B cell lymphoma cell lines and different epithelial carcinoma cell lines [21, 24]. Our experimental data suggested a correlation of the t(11;14) chromosomal translocation with Chk1 inhibitor sensitivity. A higher cytotoxicity of PF-00477736 was observed in a panel of MM cell lines displaying the t(11;14) as compared to those without the translocation and the inhibition of the CDK4/6 cyclin D1 complex activity, which in MCL cell lines is over-activated, partly neutralized the cytotoxic effect of the Chk1 inhibitor [24]. In order to better investigate the molecular markers responsible for the high response to the Chk1 inhibitor PF-00477736 of MCL cell lines, we have isolated and characterized a MCL cell line JEKO-1, resistant to the Chk1 inhibitor PF-00477736. The JEKO-1 R cell line was seven times more resistant to PF-00477736 and cross- resistant to another Chk1 inhibitor, AZD-7762, suggesting that resistance was related to the inhibition of the target. The JEKO-1 and JEKO-1 R cells were equally sensitive to bendamustine and bortezomib, two chemotherapeutic drugs commonly used in clinic for the treatment of MCL [25], and to other DNA damaging agents implying that the acquisition of the resistance to Chk1 inhibitors should not be related to resistance to other chemotherapeutic drugs. The DNA damage molecular marker γH2AX was detected in JEKO-1 R at its IC50 (150 nM) but not at 15 nM (JEKO-1 parental IC50), suggesting that this cell line acquired tolerance to treatment with the Chk1 inhibitor. In addition, analysis of DNA fragmentation and caspase-3 activation suggests that PF-00477736 resistance in JEKO-1 R is not due to inability to undergo apoptosis.

Interestingly, the JEKO-1 R cell line was also more resistant to the Wee1 inhibitor MK-1775 than the JEKO-1 parental cell line. This is an interesting and puzzling result, considering that the inhibitors are target (Chk1 or Wee1) specific [9, 29]. It could be postulated that this cross-resistance is related to the redundant roles played by Wee1 and Chk1 in both the G1-S and G2-M transitions and in ensuring a correct DNA replication [12], even if the precise molecular mechanisms are to be defined.

**Figure 6:**
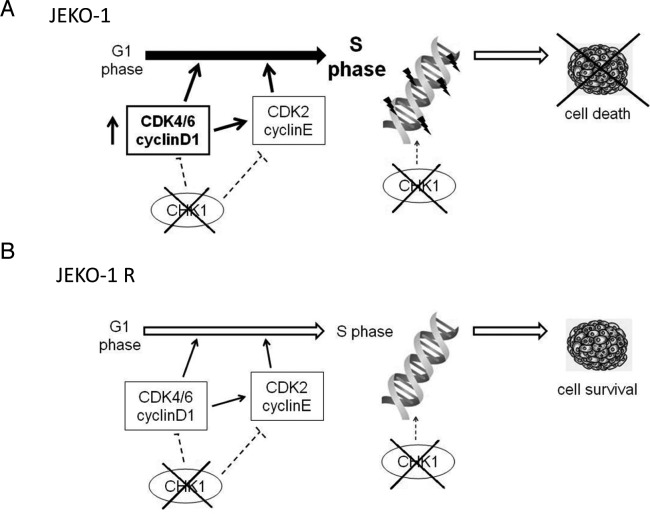
Model of Chk1 role in JEKO-1 parental and resistant cell line **A.** JEKO-1 parental cells with the chromosomal translocation t(11;14), have an enhanced G1-S transition due to cyclinD1 constitutive expression and are strongly dependent on Chk1 kinase which plays a crucial role in control of initiation of DNA replication and in the regulation of correct progression into S phase, minimizing endogenous DNA damage. Thus Chk1 inhibition in JEKO-1 cell line leads to cell death **B.**. JEKO-1 R cells showed a decrease in expression of cyclin D1 which correlates with a lower rate of G1-S transition, thus they became less dependent on Chk1 activation for a correct initiation of DNA replication and progression into S phase which proceeds quicker in the resistant cell line. Chk1 is not constitutively activated in JEKO-1 R cells and its inhibition is not lethal (see text).

When we analyzed the cell growth and cell cycle progression in parental and resistant cells, we found out that JEKO-1 R cell line has a shorter doubling time and a faster S phase than JEKO-1 parental cell line. In addition the markers of S phase, cyclin A and Cdt1, were downregulated in the JEKO-1 R cell line as compared to the parental cell line. Considering that the principal MCL molecular feature is the t(11;14), which leads to the overexpression of cyclin D1, and that our previous data suggested its involvement in the Chk1 inhibitor PF-00477736 sensitivity, we compared cyclin D1 expression in the two cell lines, and found that it was downregulated in the resistant cell line. The re-overexpression of cyclin D1 in the JEKO-1 R cell lines led to a partial restoration of the Chk1 inhibitor sensitivity, corroborating the previous hypothesis of the possible involvement of Cyclin D1 overexpression in the high responsiveness to Chk1 inhibitors. Constitutive expression of cyclin D1 leads to an enhancement of the G1-S transition, due to higher activity of the CDK4/6-cyclin D1 complex, which, by phosphorylating Rb, induces the release of E2F1 transcription factor and the consequent progression of cells into S phase [4]. The G1-S transition and the DNA replication progression is strongly controlled by Chk1 [12], possibly explaining the high activity of Chk1 inhibitors in MCL. In the resistant cells the decrease in cyclin D1 expression led to hypothesize that the activity of the CDK4/6-cyclinD1 complex is less pronounced and that the G1-S transition is less deregulated than the parental cell line (Figure 6). This is in agreement with the analysis of gene expression profile, showing that JEKO-1 R presented a lower expression of genes involved in cell cycle progression and under the control of the transcription factor E2F1 as compared to its parental cell line. Indeed, a shorter S phase and a lower Chk1 basal activity suggested that resistant cells were much less dependent on Chk1 than parental ones. These observations could also explain the increased resistance of JEKO-1 R cell line to the Wee1 inhibitor MK-1775. Wee1 plays a similar role to Chk1 in the regulation of DNA replication progression, although MCL cells rely more on Chk1 than on Wee1 activity [12, 24].

Genes belonging to pro-survival and proliferative pathways were enriched in the resistant cell line. This was somehow expected since treatment with Chk1 inhibitors leads to activation of compensatory protective pathways (MAPK, ERK) to neutralize the drugs cytotoxic effect and this might contribute to resistance to Chk1 inhibitors. Due to the observed high expression levels of FGR in JEKO-1 R, paired with additional members of the SRC pathway, we evaluated whether the inhibition of SRC pathway with a clinically available drug could overcome the resistance to PF-00477736. Treatment with the dual SRC/ABL-inhibitor dasatinib partly restored the drug sensitivity in the resistant cell line, but had no effect on parental cell line, suggesting that up-regulation of SRC and SRC-related pathways, and of downstream survival pathways, could be strictly associated with the resistance to Chk1 inhibitors in MCL. This is in agreement with data available in the literature showing *in vitro* and *in vivo* synergistic effect of Chk1 and SRC inhibitors [30, 31]. In MM it was shown that intact SRC kinase pathway is required for ERK1/2 activation and cytoprotective response after treatment with Chk1 inhibitors. On the contrary disruption of SRC kinase pathway, blocks Chk1 inhibitor induced activation of Ras-ERK1/2 signaling cascade leading to potentiation of DNA damage and activation of apoptosis. It will be worth in the future to test other drugs specifically targeting survival pathways (e.g. SRC-NFKB, RAS, MAPK pathways) in combination with Chk1 inhibitors, since these pathways may be responsible for a lower activity of Chk1 inhibitors [31]. Along with this hypothesis, our previous analysis aimed at comparing basal gene expression profile in PF-00477736 sensitive and resistant B cell lymphomas cell lines, revealed an enrichment in NFKB and JAK/STAT anti-apoptotic and pro survival pathways in the resistant ones [24].

In conclusion, the isolation and characterization of a mantle cell lymphoma cell line resistant to the Chk1 inhibitor PF-00477736 provided new mechanisms at the basis of sensitivity/resistance to the inhibition of this target. Specifically, we corroborated previous data on the involvement of the t(11;14) in the activity of Chk1 inhibitors in MCL, further supporting the preclinical evidence for the use of Chk1 inhibitors treatment as single agents in MCL. Moreover, the information gained by gene expression profile analysis suggested the possibility to apply specific drug combination to overcome resistance to Chk1 inhibitor, by interfering with pro-survival and anti-apoptotic pathways.

## MATERIALS AND METHODS

### Cell cultures and drugs

JEKO-1 and JEKO-1 R were maintained in RPMI supplemented with 1% glutamine, 1% penicillin/streptomycin and 10% fetal bovine serum (FBS). The JEKO-1 R cell line resistant to the Chk1 inhibitor PF-00477736 has been obtained from the parental cell line after continuous treatment with growing concentrations of the drug (starting from 15 nM up to 150 nM) for about one year. The lentiviral clones deriving from the JEKO-1 R cell line (control and cyclin D1) were maintained in puromycin selection at a concentration of 6 μg/ml. JEKO-1 cell line was kindly provided by Eisaku Kondo, (Okayama, JP). 293TN cell line, derived from 293 cell line, is neomycin resistant due to the presence of a neomycin resistance cassette and expressing the SV40 large T antigen, optimized for high titer production of pseudoviral particles, has been obtained from ATCC and was maintained in DMEM supplemented with 1% glutamine, and 10% fetal bovine serum (FBS). Cell line authentication was carried out by the authors within the last 6 months.

The Chk1 inhibitor PF-00477736 (Axon Medchem) was dissolved in DMSO in a stock solution of 10 mM and stored at −20°C. AZD-7762 (Selleckchem) was dissolved in DMSO in a stock solution of 20 mM. The Wee1 inhibitor MK-1775 (Chemietek) was dissolved in DMSO in a stock solution of 20 mM and stored at −20°C. Bendamustine was dissolved in DMSO in a stock solution of 200 mM and stored at −20°C. Bortezomib was dissolved in DMSO in a stock solution of 1 mM and stored at −20°C. Doxorubicine (Sigma) was dissolved in DMSO in a stock solution of 10 mM and stored at −20°C. Cis-platin (DDP) (Sigma) was dissolved in culture medium in a stock solution of 3.3 mM and stored at −20°C. Dasatinb (Axon Medchem) was dissolved in DMSO in a stock solution of 10 mM and stored at −20°C. Puromycin was dissolved in water in a stock solution of 50 μg/ml.

### Drugs treatment

Cell lines were seeded in the experimental setting of 96 well plate and treated after 48 hrs with growing concentrations of the different drugs. MTS assay, performed 72-96 hrs after treatment, was used to measure cell proliferation using a plate reader (Infinite M200, TECAN). The IC50s of the compounds investigated for the JEKO-1 and JEKO-1 R cell lines were calculated by Calcusyn Software.

### FACS-Analysis

To detect DNA by FACS, JEKO-1 and JEKO-1 R cells were fixed 24 and 48 and 72 hr after seeding and processed as already described [15]. Percentage of cell cycle phases (G1-S-G2/M) of the two cell lines was obtained analyzing DNA histograms with the previously described software [32].

### BrdUrd pulse-chase analysis

Cell cycle differences between JEKO-1 and JEKO-1 R cells were further investigated using 2′-Bromodeoxyuridine (BrdUrd) pulse-chase analysis. BrdUrd (Sigma) replaces thymidine during DNA synthesis, thus BrdUrd pulse labeling (20 min labeling with 20 μM BrdUrd added on exponential growing cells) catches S-phase cells engaged in DNA synthesis at that time [33]. Analysis at 7 hr post-labeling detects their movement through the S phase and the outflow of unlabeled cells from G1 and G2/M. The relative movement (RM), defined as the average DNA content of undivided BrdUrd-positive cells in a scale from 0 (DNA content of G1 cells) to 1 (DNA content of G2/M cells), allows us to appreciate DNA synthesis rate with the consequent evaluation of S phase duration [34]. About 2×10^6^ cells were fixed in ethanol 70% and staining and biparametric analysis were done as previously described [35]. For each sample we acquired 10,000 events with a FACS Calibur (Becton Dickinson, San Jose, CA) flow cytometer.

### Doubling time and mean phase durations

In steady state conditions a cell population grows following an exponential trend. Proliferating cells arrive in a condition of dynamic equilibrium after a short time and this condition is described by the following equation:

N(t) = N_0_ exp(ln(2)*t/T_D_)

where N(t) is the number of cells at the time t, N_0_ is the initial cell number and T_D_ represents the doubling time, i.e. the time required to the population to double the initial cell number.

Starting from the measure of the absolute cell number made by Coulter Counter (Beckman Coulter) at different times it is possible to calculate the T_D_, by fitting the growth curve with a linear regression model and calculating the angular coefficient (m) of the straight line:

ln(N(t)) = mt + ln(N_0_) with T_D_ = ln(2)/m.

Supposing that cells can proliferate without perturbations and considering negligible the intercellular variation in the phase durations, the assessment of T_D_ and the measure of cell cycle percentages (%G_1_, %S and %G_2_M), derived from flow cytometric analysis of DNA distribution or biparametric staining of BrdUrd and DNA, allows us to calculate the mean cell cycle phase durations (T_G1_, T_S_ e T_G2M_) by applying Steel's formulae [36]:

$$\begin{array}{l} {\text{T}}_{\text{G2M}}\text{=}\frac{T_{D}}{\text{In}(2)}\text{In}\left(1+\frac{\%G_{2}M}{100}\right)\\ {\text{T}}_{\text{s}}=\frac{T_{D}}{\text{In}(2)}\text{In}\left(1+\frac{\%S+\%G_{2}M}{100}\right)=T_{G2M}\\ {\text{T}}_{\text{G}1}=T_{D}-T_{s}-T_{G2M} \end{array}$$

### Caspase-3 activity assay

Caspase-3 activity was measured by enzymatic assay using a fluorogenic substrate for caspase-3, Ac-DEVD-AMC (acetyl Asp-Glu-Val-Asp 7-amido-4-methylcoumarin) as already described [21].

### Two-parameter flow cytometry analysis DNA content and FITC-conjugated dUTP

DNA fragmentation in JEKO-1 and JEKO-1 R cells treated with the same concentration and with an equitoxic dose of the Chk1 inhibitor PF-00477736 (15 nM and 150 nM respectively) was detected at 24 and 72 hr of treatment by the TdT-mediated dUTP nick-end labeling technique (TUNEL), following a procedure already described [21].

### Analysis of gene expression and real time PCR

RNA was extracted by using Maxwell 16LEV simplyRNA Cells kit (*Promega*). RNA was retro-transcribed to cDNA using High Capacity cDNA Reverse Transcription Kit (Applied Biosystem). Optimal primer pairs were chosen for each gene of interest using PRIMER-3 software (Supplementary Table 4). Differences in gene expression were determined by real time RT-PCR performed with Sybr Green PCR master mix (Applied Biosystem) and the curve of dissociation was evaluated for each gene. Samples were then normalized using the expression of the housekeeping gene (actin) and their levels were compared to control samples. Real-time PCR was done using the 7900HT Sequence Detection System (Applied Biosystems).

Gene expression profiling was done using the HumanHT-12 v4 Expression BeadChip (Illumina, San Diego, CA, USA) as previously reported [37]. Data were first extracted with the Illumina GenomeStudio software and then imported in the Partek Genomics Suite 6.4 and quantile normalized. Functional annotation was performed using the Gene Set Enrichment Analysis (GSEA) tool using the GSEA C2 and, C3.tft collections [38] and the SignatureDB gene-sets collection [39]. Raw data will be available at the National Center for Biotechnology Information Gene Expression Omnibus (http://www.ncbi.nlm.nih.gov/geo) database.

### Western blotting analysis

Proteins were extracted and visualized using standard techniques, and as already described [15]. Primary anti Chk1 (G4), cyclin D1, Cdt1, actin and Δ-tubulin were purchased from Santa Cruz Biotechnology. Primary anti pS317-Chk1, was purchased from Cell Signaling Technology. The mouse monoclonal anti-cyclin A is from BD Transduction Laboratories. The anti γH2AX antibody is from Millipore.

### Plasmid constructs

Cyclin D1 cDNA was amplified by PCR starting from JEKO-1 cDNA by using specific primers engineered to include specific restriction enzymes (EcoRI and BamHI) for subsequently cloning of the positive amplified sequence into the lentiviral vector pCDH-CMV-MCS-EF1-GFP+Puro (System Bioscience). Cloning procedure was performed with standard methodology. Primer sequences are the following: Forward: 5′ CGGAATTCATGGAACACCAGCTCCTGTGCTGCG 3′; Reverse: 5′ CGGGATCCTCAGATGTCCACGTCC CGCACGTCG 3′

### Lentiviral infection

293TN cells were co-transfected with the plasmid of interest (either control or cyclin D1) and the packaging mix of plasmids (pPACKH1-GAG, pPACKH1-REV, pVSV-G in a ratio of 5:10:6). FuGENE HD Transfection Reagent (Roche) was used. 48 hours after transfection PEG was added on the supernatant (at a ratio of 1:4) to concentrate the produced virions. This supernatant was then centrifugated and pellet containing the lentiviral particles was resuspended in PBS and was used to infect JEKO-1 R cells. The day after the infection the marker of selection puromycin was added to the cells. After some passages virus titer was evaluated by using the LV Lentiviral Titer qRT-PCR (MoBiTec) in order to be able to safely manipulate the cell culture once the titer reached non-detectable levels.

### Flow cytometric analysis of GFP content

To quantify the efficiency of the lentiviral infection % of GFP positive cells was monitored by FACS. Cells were detached and re-suspended in PBS for flow cytometric analysis. For each samples we acquired at least 10,000 events on a 256-channel scale using a FACSCalibur flow cytometer (Becton Dickinson). GFP fluorescence was detected in FL1 (530 ± 30 nm) with a logarithmic amplifier. Dead cells were excluded from the analysis by propidium iodide (PI) staining. For this purpose 1 μg/ml PI in PBS was added to cell suspensions 5 min before flow cytometric analysis. Cells could be divided into different populations: PI impermeable (viable) and PI permeable (dead cells). PI fluorescence was detected in the fluorescence channel FL3 (>670 nm) using a logarithmic amplifier. To increase the fluorescence intensity the concentration of puromycin was increased from 1 μg/ml to 6μg/ml.

## SUPPLEMENTARY MATERIAL TABLES AND FIGURES


