# Supplementary material for: Characterization of a mantle cell lymphoma cell line resistant to the Chk1 inhibitor PF-00477736
Source: Oncotarget. 2015 Oct 2;6(35):37229–40. doi: 10.18632/oncotarget.5954 (PMC4741926; doi:10.18632/oncotarget.5954)
Supplement: Supplementary file 1 [file oncotarget-06-37229-s001.pdf]

Characterization of a mantle cell lymphoma cell line resistant to the Chk1 inhibitor PF-00477736

Supplementary Material

Supplementary table 1

Summary of the IC50 for different DNA damaging agents observed in JEKO-1 and JEKO-1 R after 96 hours of treatment

Treatment	IC50 JEKO-1 (nM)	IC50 JEKO-1 R (nM)
Doxorubicin	14	16.6
5FU	10.4	12.5
Cisplatin	753.1	995.3

Supplementary Table 2

Gene sets showing enrichment (FDR < 0.05) among transcipts that were over- (2A) or down-regulated (2B) in JEKO-1 R cell line versus the JEKO-1 parental cell lin. NES, normalized enrichment score; FDR, false discovery rate.

Table 2A. Enrichement in up-regulated transcripts

NAME
CROONQUIST_NRAS_SIGNALING_UP
ICHIBA_GRAFT_VERSUS_HOST_DISEASE_35D_UP
GAURNIER_PSMD4_TARGETS
WIELAND_UP_BY_HBV_INFECTION
WALLACE_PROSTATE_CANCER_RACE_UP
BROWN_MYELOID_CELL_DEVELOPMENT_UP
DIRMEIER_LMP1_RESPONSE_EARLY
NIKOLSKY_BREAST_CANCER_17Q11_Q21_AMPLICON
WUNDER_INFLAMMATORY_RESPONSE_AND_CHOLESTEROL_UP
IKEDA_MIR1_TARGETS_UP
FLECHNER_BIOPSY_KIDNEY_TRANSPLANT_REJECTED_VS_OK_UP
BOYLAN_MULTIPLE_MYELOMA_PCA1_UP
JAATINEN_HEMATOPOIETIC_STEM_CELL_DN
RODWELL_AGING_KIDNEY_UP
ZHAN_V1_LATE_DIFFERENTIATION_GENES_UP
KIM_GLIS2_TARGETS_UP
QI_PLASMACYTOMA_UP
MCLACHLAN_DENTAL_CARIES_UP
CROONQUIST_NRAS_VS_STROMAL_STIMULATION_UP
HESS_TARGETS_OF_HOXA9_AND_MEIS1_DN
PELLICCIOTTA_HDAC_IN_ANTIGEN_PRESENTATION_UP
VERRECCHIA_EARLY_RESPONSE_TO_TGFB1

REN_ALVEOLAR_RHABDOMYOSARCOMA_DN

BASSO_CD40_SIGNALING_UP

HOSHIDA_LIVER_CANCER_SUBCLASS_S1

LEE_DIFFERENTIATING_T_LYMPHOCYTE

WINTER_HYPOXIA_DN

MORI_LARGE_PRE_BII_LYMPHOCYTE_DN

FARMER_BREAST_CANCER_CLUSTER_1

BILBAN_B_CLL_LPL_DN

HUNSBERGER_EXERCISE_REGULATED_GENES

BOYAULT_LIVER_CANCER_SUBCLASS_G5_DN

VILIMAS_NOTCH1_TARGETS_UP

SMID_BREAST_CANCER_NORMAL_LIKE_UP

RODRIGUES_THYROID_CARCINOMA_DN

HOLLMANN_APOPTOSIS_VIA_CD40_UP

ZHENG_BOUND_BY_FOXP3

AMIT_SERUM_RESPONSE_40_MCF10A

LINDSTEDT_DENDRITIC_CELL_MATURATION_D

DING_LUNG_CANCER_EXPRESSION_BY_COPY_NUMBER

WU_SILENCED_BY_METHYLATION_IN_BLADDER_CANCER

BOYLAN_MULTIPLE_MYELOMA_C_D_DN

SHAFFER_IRF4_MULTIPLE_MYELOMA_PROGRAM

LU_IL4_SIGNALING

HUANG_DASATINIB_RESISTANCE_UP

NAGASHIMA_NRG1_SIGNALING_UP

SANA_RESPONSE_TO_IFNG_UP

ZHENG_FOXP3_TARGETS_IN_THYMUS_UP

LEIN_LOCALIZED_TO_DISTAL_AND_PROXIMAL_DENDRITES

AZARE_NEOPLASTIC_TRANSFORMATION_BY_STAT3_DN

SCHUETZ_BREAST_CANCER_DUCTAL_INVASIVE_UP

DER_IFN_BETA_RESPONSE_UP

RAMALHO_STEMNESS_DN

CROMER_TUMORIGENESIS_DN

NAKAYAMA_SOFT_TISSUE_TUMORS_PCA1_UP

SCHURINGA_STAT5A_TARGETS_DN

LIU_VAV3_PROSTATE_CARCINOGENESIS_UP

POOLA_INVASIVE_BREAST_CANCER_UP

DER_IFN_ALPHA_RESPONSE_UP

LEE_INTRATHYMIC_T_PROGENITOR

NIKOLSKY_BREAST_CANCER_16P13_AMPLICON

SPIRA_SMOKERS_LUNG_CANCER_UP

HOFFMANN_PRE_BI_TO_LARGE_PRE_BII_LYMPHOCYTE_UP

CHEN_ETV5_TARGETS_SERTOLI

MISHRA_CARCINOMA_ASSOCIATED_FIBROBLAST_UP

TARTE_PLASMA_CELL_VS_B_LYMPHOCYTE_UP

HAHTOLA_MYCOSIS_FUNGOIDES_DN

TAKEDA_TARGETS_OF_NUP98_HOXA9_FUSION_6HR_UP

FERRANDO_T_ALL_WITH_MLL_ENL_FUSION_UP

VERRECCHIA_RESPONSE_TO_TGFB1_C2

DEBOSSCHER_NFKB_TARGETS_REPRESSED_BY_GLUCOCORTICOIDS

BURTON_ADIPOGENESIS_9

KUROZUMI_RESPONSE_TO_ONCOCYTIC_VIRUS

ROZANOV_MMP14_TARGETS_UP

KORKOLA_TERATOMA_UP

MCLACHLAN_DENTAL_CARIES_DN

NIELSEN_SYNOVIAL_SARCOMA_DN

GALE_APL_WITH_FLT3_MUTATED_UP

HOSHIDA_LIVER_CANCER_LATE_RECURRENCE_UP

BOSCO_TH1_CYTOTOXIC_MODULE

NADLER_OBESITY_UP

LIAN_LIPA_TARGETS_3M

THUM_SYSTOLIC_HEART_FAILURE_UP

WANG_RESPONSE_TO_GSK3_INHIBITOR_SB216763_UP

BOYAULT_LIVER_CANCER_SUBCLASS_G6_DN

BRIDEAU_IMPRINTED_GENES

KLEIN_PRIMARY_EFFUSION_LYMPHOMA_DN

FIGUEROA_AML_METHYLATION_CLUSTER_5_DN

GAVIN_FOXP3_TARGETS_CLUSTER_P3

PARK_APL_PATHOGENESIS_DN

HASLINGER_B_CLL_WITH_CHROMOSOME_12_TRISOMY

ODONNELL_TARGETS_OF_MYC_AND_TFRC_UP

NAGASHIMA_EGF_SIGNALING_UP

LIAN_LIPA_TARGETS_6M

YOKOE_CANCER_TESTIS_ANTIGENS

ICHIBA_GRAFT_VERSUS_HOST_DISEASE_D7_UP

THEILGAARD_NEUTROPHIL_AT_SKIN_WOUND_DN

TAVOR_CEBPA_TARGETS_UP

CHEN_LIVER_METABOLISM_QTL_CIS

RUTELLA_RESPONSE_TO_HGF_DN

NIKOLSKY_BREAST_CANCER_12Q13_Q21_AMPLICON

FURUKAWA_DUSP6_TARGETS_PCI35_UP

CHAUHAN_RESPONSE_TO_METHOXYESTRADIOL_DN

GRAHAM_CML_QUIESCENT_VS_NORMAL_QUIESCENT_DN

WIERENGA_STAT5A_TARGETS_GROUP2

LANDIS_ERBB2_BREAST_PRENEOPLASTIC_DN

SANSOM_APC_TARGETS_DN

FIGUEROA_AML_METHYLATION_CLUSTER_6_DN

ZHAN_MULTIPLE_MYELOMA_HP_DN

IIZUKA_LIVER_CANCER_PROGRESSION_L0_L1_DN

FLECHNER_PBL_KIDNEY_TRANSPLANT_OK_VS_DONOR_DN

BROWNE_INTERFERON_RESPONSIVE_GENES

FOSTER_TOLERANT_MACROPHAGE_UP

MARKEY_RB1_ACUTE_LOF_UP

RUTELLA_RESPONSE_TO_CSF2RB_AND_IL4_DN

BROWNE_HCMV_INFECTION_4HR_UP

LE_NEURONAL_DIFFERENTIATION_UP

DAUER_STAT3_TARGETS_DN

TAKEDA_TARGETS_OF_NUP98_HOXA9_FUSION_3D_UP

FAELT_B_CLL_WITH_VH_REARRANGEMENTS_UP

PHONG_TNF_TARGETS_UP

TSAI_RESPONSE_TO_RADIATION_THERAPY

SCHAEFFER_PROSTATE_DEVELOPMENT_6HR_UP

WANG_ESOPHAGUS_CANCER_VS_NORMAL_UP

IKEDA_MIR30_TARGETS_UP

SHAFFER_IRF4_TARGETS_IN_MYELOMA_VS_MATURE_B_LYMPHOCYTE

TONKS_TARGETS_OF_RUNX1_RUNX1T1_FUSION_HSC_DN

MARTORIATI_MDM4_TARGETS_NEUROEPITHELIUM_DN

PASQUALUCCI_LYMPHOMA_BY_GC_STAGE_DN

DAVIES_MULTIPLE_MYELOMA_VS_MGUS_DN

COLLIS_PRKDC_SUBSTRATES

ASTIER_INTEGRIN_SIGNALING

RASHI_RESPONSE_TO_IONIZING_RADIATION_6

BEIER_GLIOMA_STEM_CELL_DN

LI_INDUCED_T_TO_NATURAL_KILLER_UP

VALK_AML_CLUSTER_5

DIAZ_CHRONIC_MEYLOGENOUS_LEUKEMIA_DN

GAVIN_PDE3B_TARGETS

MORI_MATURE_B_LYMPHOCYTE_UP

BILBAN_B_CLL_LPL_UP

SWEET_KRAS_TARGETS_UP

MIKKELSEN_MEF_LCP_WITH_H3K4ME3

LINDGREN_BLADDER_CANCER_CLUSTER_2B

TOOKER_GEMCITABINE_RESISTANCE_UP

VANASSE_BCL2_TARGETS_UP

YAN_ESCAPE_FROM_ANOIKIS

SENGUPTA_EBNA1_ANTICORRELATED

MATSUDA_NATURAL_KILLER_DIFFERENTIATION

YU_MYC_TARGETS_DN

GRAESSMANN_RESPONSE_TO_MC_AND_SERUM_DEPRIVATION_DN

MASRI_RESISTANCE_TO_TAMOXIFEN_AND_AROMATASE_INHIBITORS_DN

NUTT_GBM_VS_AO_GLIOMA_UP

TURASHVILI_BREAST_LOBULAR_CARCINOMA_VS_DUCTAL_NORMAL_UP

PETROVA_PROX1_TARGETS_DN

KYNG_DNA_DAMAGE_BY_GAMMA_RADIATION

DAZARD_RESPONSE_TO_UV_SCC_UP

GARGALOVIC_RESPONSE_TO_OXIDIZED_PHOSPHOLIPIDS_TURQUOISE_UP

HOFMANN_MYELODYSPLASTIC_SYNDROM_RISK_DN

ONO_AML1_TARGETS_UP

CROMER_METASTASIS_UP

BASSO_HAIRY_CELL_LEUKEMIA_DN

FULCHER_INFLAMMATORY_RESPONSE_LECTIN_VS_LPS_DN

ZHENG_GLIOBLASTOMA_PLASTICITY_DN

AMIT_EGF_RESPONSE_60_MCF10A

SHEDDEN_LUNG_CANCER_GOOD_SURVIVAL_A12

PICCALUGA_ANGIOIMMUNOBLASTIC_LYMPHOMA_DN

OSMAN_BLADDER_CANCER_UP

MORI_SMALL_PRE_BII_LYMPHOCYTE_DN

APPEL_IMATINIB_RESPONSE

MORI_IMMATURE_B_LYMPHOCYTE_UP

MUNSHI_MULTIPLE_MYELOMA_UP

BAUS_TFF2_TARGETS_UP

LIU_IL13_PRIMING_MODEL

HIRSCH_CELLULAR_TRANSFORMATION_SIGNATURE_UP

TAKEDA_TARGETS_OF_NUP98_HOXA9_FUSION_8D_UP

SEKI_INFLAMMATORY_RESPONSE_LPS_UP

BARRIER_COLON_CANCER_RECURRENCE_UP

ROSS_AML_WITH_MLL_FUSIONS

KIM_LRRC3B_TARGETS

CLIMENT_BREAST_CANCER_COPY_NUMBER_UP

KUUSELO_PANCREATIC_CANCER_19Q13_AMPLIFICATION

STEARMAN_TUMOR_FIELD_EFFECT_UP

WIERENGA_STAT5A_TARGETS_UP

BOYAULT_LIVER_CANCER_SUBCLASS_G123_UP

EBAUER_MYOGENIC_TARGETS_OF_PAX3_FOXO1_FUSION

AKL_HTLV1_INFECTION_DN

KOBAYASHI_RESPONSE_TO_ROMIDEPSIN

PETRETTO_HEART_MASS_QTL_CIS_DN

JAZAG_TGFB1_SIGNALING_UP

KUNINGER_IGF1_VS_PDGFB_TARGETS_UP

ZHAN_MULTIPLE_MYELOMA_LB_DN

DANG_MYC_TARGETS_DN

DUNNE_TARGETS_OF_AML1_MTG8_FUSION_DN

MILI_PSEUDOPODIA_CHEMOTAXIS_DN

DEURIG_T_CELL_PROLYMPHOCYTIC_LEUKEMIA_DN

TIAN_TNF_SIGNALING_NOT_VIA_NFKB

SUNG_METASTASIS_STROMA_UP

CHIBA_RESPONSE_TO_TSA_DN

PLASARI_NFIC_TARGETS_BASAL_DN

DORSEY_GAB2_TARGETS

LENAOUR_DENDRITIC_CELL_MATURATION_DN

CHEMELLO_SOLEUS_VS_EDL_MYOFIBERS_DN

ALTEMEIER_RESPONSE_TO_LPS_WITH_MECHANICAL_VENTILATION

VETTER_TARGETS_OF_PRKCA_AND_ETS1_UP

ZHENG_FOXP3_TARGETS_UP

KUROZUMI_RESPONSE_TO_ONCOCYTIC_VIRUS_AND_CYCLIC_RGD

RUTELLA_RESPONSE_TO_HGF_VS_CSF2RB_AND_IL4_UP

GESERICK_TERT_TARGETS_DN

OSWALD_HEMATOPOIETIC_STEM_CELL_IN_COLLAGEN_GEL_DN

MAGRANGEAS_MULTIPLE_MYELOMA_IGLL_VS_IGLK_UP

NIELSEN_LEIOMYOSARCOMA_UP

ACEVEDO_NORMAL_TISSUE_ADJACENT_TO_LIVER_TUMOR_UP

HAHTOLA_MYCOSIS_FUNGOIDES_SKIN_UP

MCBRYAN_PUBERTAL_TGFB1_TARGETS_DN

XU_CREBBP_TARGETS_DN

POTTI_ETOPOSIDE_SENSITIVITY

WANG_TARGETS_OF_MLL_CBP_FUSION_DN

HASLINGER_B_CLL_WITH_11Q23_DELETION

BERENJENO_TRANSFORMED_BY_RHOA_REVERSIBLY_DN

DEMAGALHAES_AGING_UP

XU_RESPONSE_TO_TRETINOIN_AND_NSC682994_UP

WEIGEL_OXIDATIVE_STRESS_BY_TBH_AND_H2O2

FINAK_BREAST_CANCER_SDPP_SIGNATURE

MORI_PRE_BI_LYMPHOCYTE_DN

MISHRA_CARCINOMA_ASSOCIATED_FIBROBLAST_DN

IKEDA_MIR133_TARGETS_UP

LIU_SMARCA4_TARGETS

GAVIN_FOXP3_TARGETS_CLUSTER_P7

KAAB_FAILED_HEART_ATRIUM_DN

PARK_HSC_AND_MULTIPOTENT_PROGENITORS

MONNIER_POSTRADIATION_TUMOR_ESCAPE_DN

UROSEVIC_RESPONSE_TO_IMIQUIMOD

BIOCARTA_CTLA4_PATHWAY

BIOCARTA_IGF1_PATHWAY

BIOCARTA_INSULIN_PATHWAY

BIOCARTA_NO2IL12_PATHWAY

BIOCARTA_IL12_PATHWAY

BIOCARTA_MAL_PATHWAY

BIOCARTA_MET_PATHWAY

BIOCARTA_PTEN_PATHWAY

BIOCARTA_HCMV_PATHWAY

BIOCARTA_SPRY_PATHWAY

BIOCARTA_BARRESTIN_SRC_PATHWAY

BIOCARTA_PDGF_PATHWAY

BIOCARTA_TOLL_PATHWAY

BIOCARTA_AT1R_PATHWAY

KEGG_INTESTINAL_IMMUNE_NETWORK_FOR_IGA_PRODUCTION

KEGG_ALLOGRAFT_REJECTION

KEGG_GRAFT_VERSUS_HOST_DISEASE

KEGG_TYPE_I_DIABETES_MELLITUS

KEGG_AUTOIMMUNE_THYROID_DISEASE

KEGG_ASTHMA

KEGG_CYTOSOLIC_DNA_SENSING_PATHWAY

KEGG_CHEMOKINE_SIGNALING_PATHWAY

KEGG_LEISHMANIA_INFECTION

KEGG_CELL_ADHESION_MOLECULES_CAMS

KEGG_VIRAL_MYOCARDITIS

KEGG_ANTIGEN_PROCESSING_AND_PRESENTATION

KEGG_LEUKOCYTE_TRANSENDOTHELIAL_MIGRATION

KEGG_TOLL_LIKE_RECEPTOR_SIGNALING_PATHWAY

KEGG_STARCH_AND_SUCROSE_METABOLISM

KEGG_ALDOSTERONE_REGULATED_SODIUM_REABSORPTION

KEGG_REGULATION_OF_ACTIN_CYTOSKELETON

KEGG_PRIMARY_IMMUNODEFICIENCY

KEGG_ENDOMETRIAL_CANCER

KEGG_T_CELL_RECEPTOR_SIGNALING_PATHWAY

KEGG_VASCULAR_SMOOTH_MUSCLE_CONTRACTION

KEGG_FOCAL_ADHESION

KEGG_NOD_LIKE_RECEPTOR_SIGNALING_PATHWAY

KEGG_PATHOGENIC_ESCHERICHIA_COLI_INFECTION

KEGG_TIGHT_JUNCTION

KEGG_EPITHELIAL_CELL_SIGNALING_IN_HELICOBACTER_PYLORI_INFECTION

KEGG_NATURAL_KILLER_CELL_MEDIATED_CYTOTOXICITY

KEGG_CYTOKINE_CYTOKINE_RECEPTOR_INTERACTION

KEGG_RENAL_CELL_CARCINOMA

REACTOME_IMMUNOREGULATORY_INTERACTIONS_BETWEEN_A_LYMPHOID_AND_A_NON_LYMPHOID_CELL

REACTOME_DOWNSTREAM_TCR_SIGNALING

REACTOME_GENERATION_OF_SECOND_MESSENGER_MOLECULES

REACTOME_TCR_SIGNALING

REACTOME_MYOGENESIS

REACTOME_PHOSPHORYLATION_OF_CD3_AND_TCR_ZETA_CHAINS

REACTOME_CHEMOKINE_RECEPTORS_BIND_CHEMOKINES

REACTOME_PD1_SIGNALING

REACTOME_ACTIVATED_TLR4_SIGNALLING

REACTOME_MYD88_MAL_CASCADE_INITIATED_ON_PLASMA_MEMBRANE

REACTOME_TOLL_RECEPTOR_CASCADES

REACTOME_TRIF_MEDIATED_TLR3_SIGNALING

REACTOME_NFKB_AND_MAP_KINASES_ACTIVATION_MEDIATED_BY_TLR4_SIGNALING_REPERTOIRE

REACTOME_INTERFERON_GAMMA_SIGNALING

REACTOME_MAP_KINASE_ACTIVATION_IN_TLR_CASCADE

RESPONSE_TO_VIRUS

RESPONSE_TO_OTHER_ORGANISM

CALCIUM_ION_BINDING

CAMP_UP.V1_DN

GLI1_UP.V1_UP

RPS14_DN.V1_UP

SNF5_DN.V1_UP

STK33_UP

GLI1_UP.V1_DN

ESC_J1_UP_EARLY.V1_DN

STK33_SKM_UP

STK33_NOMO_UP

ERB2_UP.V1_DN

JNK_DN.V1_DN

RB_P130_DN.V1_DN

PIGF_UP.V1_UP

LTE2_UP.V1_UP

KRAS.LUNG_UP.V1_UP

BLIMP_BCELL_REPRESSED

HEMATOPOIETIC_NODE1658

RESTING_DENDRITIC_CELL_GNF

TCELL_PIREP_CSAUP4X

IRF4_MYELOMA_INDUCED_DIRECT

CD40_UPREGULATED_BURKITT_LYMPHOMA

TCELL_PIREP_CALCIUMDEFPTUP4X_FESKE_FIG6

PLASMA_CELL_GT_MATURE_BCELL

STAT3_UP_OCILY10

PAX5_REPRESSED

GERMINAL_CENTER_B_CELL_DLBCL_SURVIVAL_PREDICTOR

CC_1P5_UP

MYELOMA_HY_SUBGROUP_DOWN

MYD88_ALL_DOWN

BLOOD_MODULE-1.5_MYELOID_LINEAGE-1

QUIESCENCE_HEME_CLUSTER1

BLOOD_MODULE-3.3_INFLAMMATION-2

CLL_MUTATED_GT_CLL_UNMUTATED

QUIESCENCE_HEME_ALL

BLOOD_CD4+TCELL_GT_THYMIC_SP_CD4+TCELL

RESTING_BLOOD_B_CELL_GNF

IFN_PMBC_4X_UP

NFKB_UP_ALL_OCILY3_LY10

BLOOD_MODULE-2.1_CYTOTOXIC_CELLS

JAK_IL10_LY10_UP

KRAS_UP

CC_1P33_UP

DENDRITIC_CELL_CD123POS_BLOOD

MYELOID_NODE1536

BLOOD_MODULE-1.3_B_CELLS

NFKB_UP_K1106

LYMPH_NODE_DLBCL

IRF4_MYELOMA_INDUCED_LYMPHOCHIP

IFN_PMBC_2X_UP

T_NK_NODE1604

MYELOMA_TACI_HIGH_BONE_MARROW_PLASMA_CELL_GENE

PI3K_OVEREXPRESSION_DOWN

GERMINAL_CENTER_BCELL_DLBCL

MYC_CHIP_PET_EXPR_DOWN

SPLENIC_MARGINAL_ZONE_BCELL_GT_GC_BCELL

GC_T_HELPER_UP2X_CHTANOVA

MCL_GT_SLL_DLBCL

IL10_OCILY3_UP

NFKB_UP_OCILY10_ONLY

TCELL_CYTOKINE_INDUCED_PMBC_BCELL_INDUCED

BLOOD_MODULE-3.9_UNDETERMINED

THYMIC_DP_TCELL_GT_THYMIC_PROGENITOR_TCELL

MYELOMA_LB_SUBGROUP_DOWN

IRF4_TARGET_MM_PLASMA_CELL_GT_B_CELL

BLOOD_MODULE-3.4_UNDETERMINED

B_CELL_UP_ANERGY

THYMIC_SP_CD4+TCELL_GT_BLOOD_CD4+TCELL

TCELL_PIIND4X_CSAINDEPENDENT

TCELL_PIIND4X_FESKE_FIG4

DENDRITIC_CELL_CD16POS_BLOOD

GC_B_CELL_BL_EQUAL_DLBCL

BLOOD_MODULE-3.1_INTERFERON_INDUCIBLE

NFKB_UP_BOTHOCILY3ANDLY10

HRAS_OVEREXPRESSION_4X_UP

BLOOD_MODULE-3.2_INFLAMMATION-1

STAT3HIGH_ABC_DLBCL_SUBGROUP

LEUCINE_STARVE_DOWN

IRF3_TARGET_GENE

BCL6_TARGETS_CHIPCHIP

DENDRITIC_CELL_BDCA3POS_BLOOD

Table 2B. Enrichement in down-regulated transcripts

NAME
LINDGREN_BLADDER_CANCER_WITH_LOH_IN_CHR9Q
KRIGE_AMINO_ACID_DEPRIVATION
RODRIGUES_NTN1_TARGETS_UP
POMEROY_MEDULLOBLASTOMA_PROGNOSIS_DN
REN_BOUND_BY_E2F
TAKADA_GASTRIC_CANCER_COPY_NUMBER_DN
KEGG_RIBOSOME
REACTOME_MEIOTIC_SYNAPSIS
REACTOME_TELOMERE_MAINTENANCE
REACTOME_PACKAGING_OF_TELOMERE_ENDS
REACTOME_DEPOSITION_OF_NEW_CENPA_CONTAINING_NUCLEOSOMES_AT_THE_CENTROMERE
REACTOME_RNA_POL_I_PROMOTER_OPENING
REACTOME_CHROMOSOME_MAINTENANCE
REACTOME_MEIOSIS
REACTOME_MEIOTIC_RECOMBINATION
REACTOME_RNA_POL_I_TRANSCRIPTION
REACTOME_AMYLOIDS
REACTOME_RNA_POL_I_RNA_POL_III_AND_MITOCHONDRIAL_TRANSCRIPTION
REACTOME_PEPTIDE_CHAIN_ELONGATION
REACTOME_TRANSCRIPTION
REACTOME_CELL_CYCLE
REACTOME_EARLY_PHASE_OF_HIV_LIFE_CYCLE
REACTOME_INFLUENZA_VIRAL_RNA_TRANSCRIPTION_AND_REPLICATION

REACTOME_AMINO_ACID_SYNTHESIS_AND_INTERCONVERSION_TRANSAMINATION

REACTOME_YAP1_AND_WWTR1_TAZ_STIMULATED_GENE_EXPRESSION

REACTOME_INFLUENZA_LIFE_CYCLE

MITOCHONDRIAL_RESPIRATORY_CHAIN

STRUCTURAL_CONSTITUENT_OF_RIBOSOME

RPS14_DN.V1_DN

PDGF_ERK_DN.V1_DN

BLOOD_MODULE-2.4_RIBOSOMAL

MYC_CHIP_PET_EXPR_UP

BLOOD_MODULE-1.7_MHC_RIBOSOMAL_PROTEINS

PGC-1ALPHA_OVEREPXRESSION_UP

LEUCINE_STARVE_UP

Supplementary Table 3

Top-50 up- and top 50 most down-ranked transcripts according to GSEA in JEKO-1 R cell line in comparison with its parental cell line.

Gene Symbol	GSEA SCORE	Fold-Change	t-test P-value
FGR	0.68	5.12	3.51E-04
SYT11	0.60	4.28	3.51E-05
RAB17	0.60	3.60	4.82E-02
FCRL2	0.57	1.94	1.20E-02
CCL3L3	0.54	3.26	7.29E-04
LOC728835	0.54	3.28	1.93E-03
IGJ	0.52	4.16	2.93E-02
CCL4L1	0.49	2.84	3.62E-03
CCL4L2	0.48	2.40	1.20E-02
SBK1	0.46	2.83	2.06E-03
RGS1	0.45	2.52	2.00E-02
ACTG2	0.44	2.52	n.s.
CD3D	0.43	1.03	n.s.
CREB5	0.43	2.54	7.59E-03
APOC2	0.43	2.49	1.77E-02
SPRED1	0.40	2.27	1.54E-02
STAP1	0.38	2.26	7.55E-03
SPINT2	0.38	2.30	3.49E-03
FCRL5	0.37	1.33	5.41E-03
PARN	0.37	2.50	9.33E-03
LOC100133583	0.37	2.41	3.49E-02
FAM177B	0.36	2.12	1.64E-02

TNFRSF17	0.35	2.22	3.04E-02
LOC100129076	0.35	2.06	2.84E-02
CEACAM1	0.35	1.76	1.93E-03
MGC4677	0.34	2.18	5.27E-05
PLEKHO1	0.34	2.27	1.35E-02
TUBB2B	0.34	2.71	n.s.
TCTEX1D2	0.34	2.28	3.32E-03
IRAK3	0.34	1.98	7.18E-03
CPLX1	0.33	1.99	n.s.
VAV3	0.33	1.95	6.12E-04
MTSS1	0.33	2.23	n.s.
TSPAN32	0.33	1.95	1.52E-02
C11ORF17	0.33	2.13	3.55E-02
TMPRSS3	0.33	1.94	1.88E-02
SP110	0.33	2.13	1.28E-02
HLA-DQB1	0.32	1.99	1.05E-02
ARHGEF6	0.32	2.20	4.68E-02
PTK7	0.32	1.93	1.26E-02
HLA-DOB	0.32	2.01	1.94E-03
TYROBP	0.31	2.02	2.79E-02
TPM2	0.31	2.22	4.56E-02
FCRL3	0.31	1.86	3.72E-03
CXORF48	0.31	1.30	4.05E-03
C3ORF37	0.30	2.02	9.48E-03
ASB10	0.30	1.79	3.90E-02
PCDH9	0.30	1.81	n.s.
MXD4	0.30	1.97	n.s.

LOC221442	0.30	1.83	1.19E-02
SNORA12	-0.92	-8.70	6.46E-04
SCARNA11	-0.69	-4.38	1.29E-02
SCARNA13	-0.69	-4.40	3.10E-03
CDKN1A	-0.66	-4.86	1.22E-02
SCARNA23	-0.59	-3.38	n.s.
SCARNA8	-0.57	-3.13	3.07E-02
SLC2A5	-0.55	-3.73	1.15E-02
SCARNA16	-0.53	-3.11	n.s.
FAM38A	-0.53	-3.17	3.18E-05
SNORA79	-0.52	-2.83	2.14E-02
BMP7	-0.49	-3.12	n.s.
RMRP	-0.49	-2.70	n.s.
SCARNA14	-0.47	-2.60	2.16E-03
TMEM100	-0.47	-2.93	3.62E-02
NETO2	-0.46	-2.45	1.60E-03
COBLL1	-0.46	-2.42	4.85E-02
BBOX1	-0.45	-2.45	3.29E-02
GNPDA1	-0.44	-2.73	1.22E-03
HOMO SAPIENS CLONE 2	-0.44	n.a.	n.a.
PDE9A	-0.42	-2.52	3.63E-02
TEAD2	-0.40	-2.36	4.71E-02
VTRNA1-1	-0.36	-2.02	1.54E-02
SNTB1	-0.36	-2.08	n.s.
HIST1H2BD	-0.35	-2.55	2.77E-02
SNORD13	-0.35	-2.28	n.s.
RNY1	-0.35	-2.11	n.s.

PKN3	-0.34	-2.03	1.70E-02
OSBPL10	-0.34	-2.09	4.15E-02
CCNA2	-0.34	-2.50	5.50E-03
SNHG8	-0.34	-2.55	8.68E-03
WNT5A	-0.34	-1.97	4.92E-02
TERC	-0.34	-1.93	5.00E-02
SNORD3A	-0.34	-2.28	n.s.
HIST1H2BK	-0.33	-3.55	n.s.
LOC100130592	-0.31	-1.85	n.s.
SNORD3C	-0.31	-1.94	n.s.
SNORA63	-0.31	-1.89	n.s.
ADARB1	-0.30	-1.86	n.s.
HIST1H2BJ	-0.30	-2.01	n.s.
TRIB2	-0.30	-1.82	n.s.
HUMAN SMALL NUCLEAR	-0.30	n.a.	n.a.
SNORA27	-0.30	-1.83	1.71E-02
HIST2H2BE	-0.30	-1.96	n.s.
ECH1	-0.30	-1.92	n.s.
LOC100132439	-0.29	-1.77	2.69E-02
KCNQ2	-0.29	-2.01	4.29E-02
NMT2	-0.29	-1.91	n.s.
LRRC26	-0.29	-1.82	1.23E-02
NFATC1	-0.29	-1.92	2.31E-02
SNORD3D	-0.29	-2.07	n.s.

Supplementary table 4

List of primers used for real-time PCR on cDNA

	Primer FOR	Primer REV
ß-actin	TACAATGAGCTGCGTGTGG	GGGGTGTTGAAGGTCTCAAA
ABCB1	CCCATCATTGCAATAGCAGG	GTTCAAACTTCTGCTCCTGA
ABCC1	TCACAGGGTTGATTGTCCGG	ACTTGTTCCGACGTGTCCTC
ABCG2	AGGTGGAGGCAAATCTTCGTT	TCGCGGTGCTCCATTTATCA
Cdt1	GGAGAAGCTCACCACTGCTC	TGACTCAAGGCCTTCTCCAT
Cyclin D1	CCCTCGGTGTCCTATTCAA	AAGACCTCCTCCTCGCACTT
Cyclin A	CCACAGCATGCACAACAGTC	GTGTCTCTGGTGGGTTGAGG

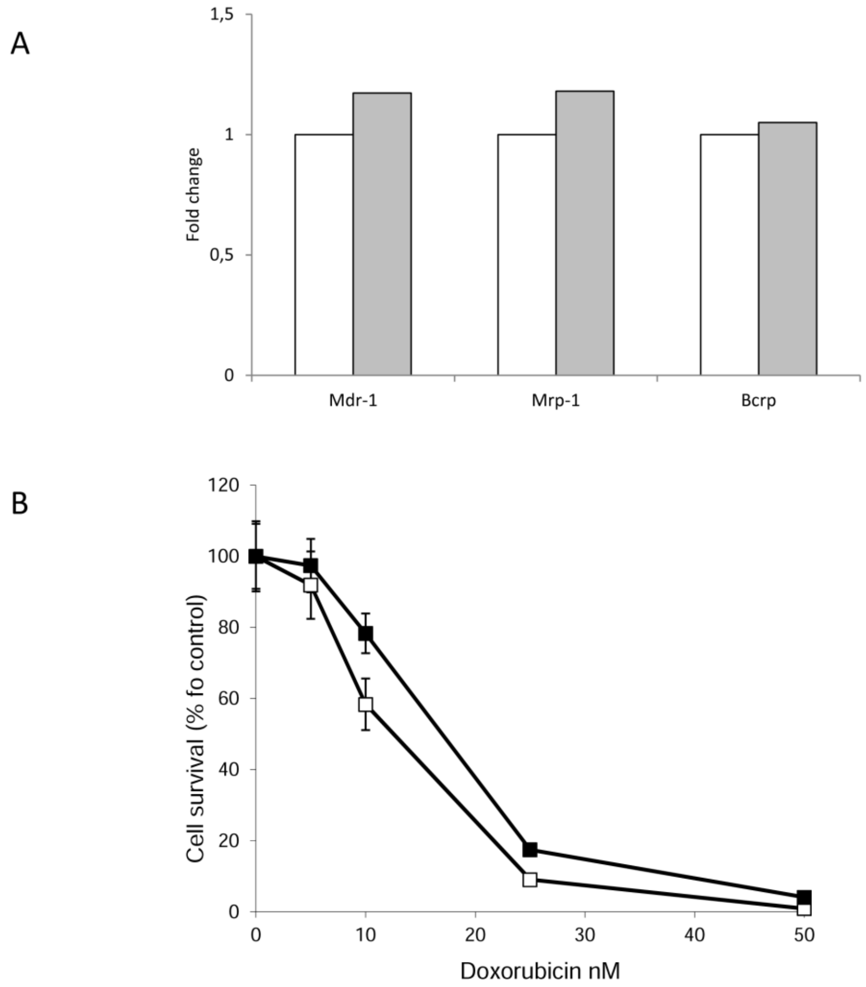

Supplementary figure 1

(A) Real time PCR showing Mdr-1, Mrp-1 and Bcrp expression levels in parental JEKO-1 cells (white bar) and in JEKO-1 R cells (grey bar). Data are normalized to the internal mRNA levels of actin and are represented as the fold change from JEKO-1 parental samples.

(B) Cytotoxic effect of Doxorubicine in JEKO-1 parental (■) and in JEKO-1 R (□) cells

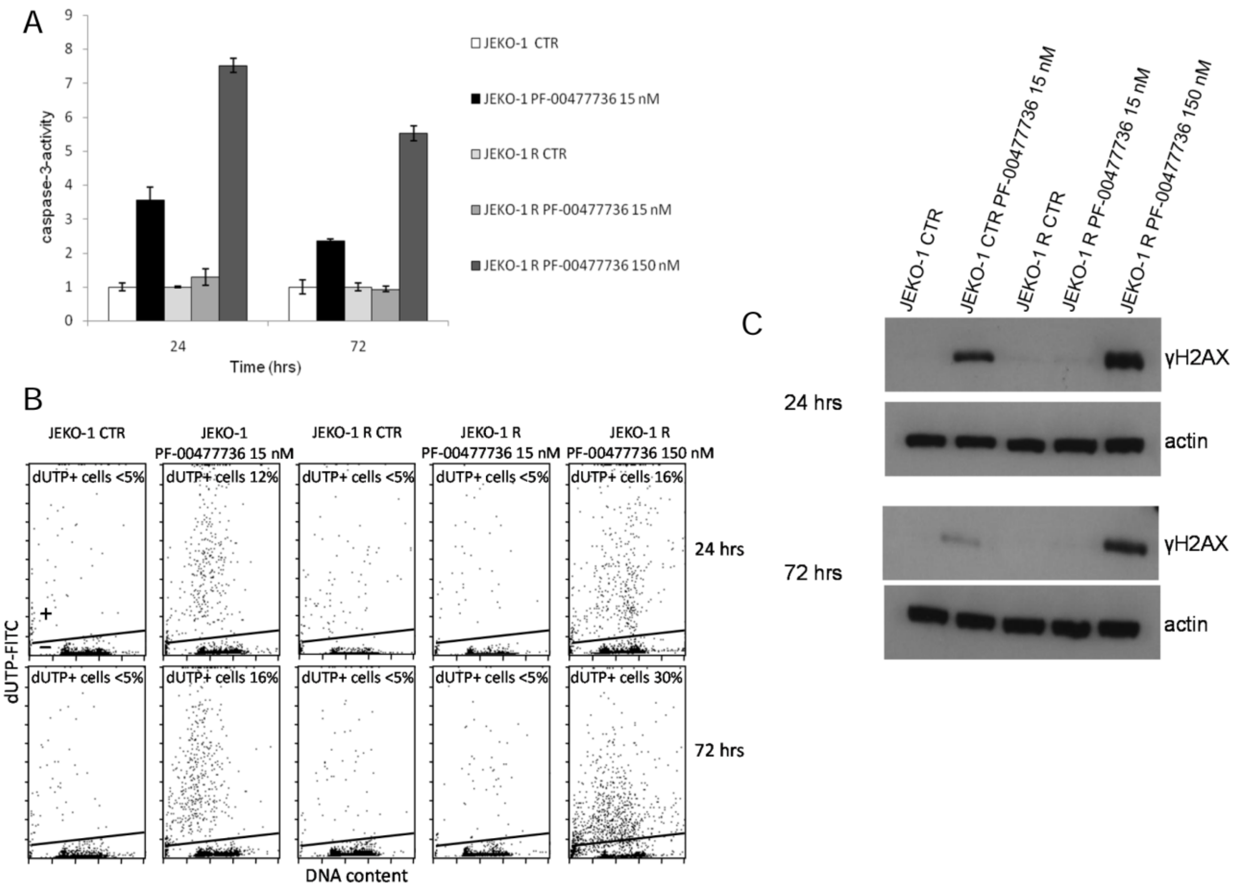

Supplementary Figure 2

(A) Activation of caspase-3 by enzymatic assay in JEKO-1 parental and JEKO-1 R cells at 24, and 72 hrs after treatment with PF-00477736 at equimolar concentrations 15 nM (both) and at equitoxic concentrations (IC50: 15 nM for JEKO-1 and 150 nM for JEKO-1 R). Data are represented as fold change over untreated cells and are the mean ±SD of two independent experiments.

(B) TUNEL assay performed in JEKO-1 parental and JEKO-1 R cells at the same experimental conditions as above

(C) Western blot analysis showing γH2AX and actin protein levels in JEKO-1 parental and JEKO-1 R treated at the same experimental conditions as above specified.

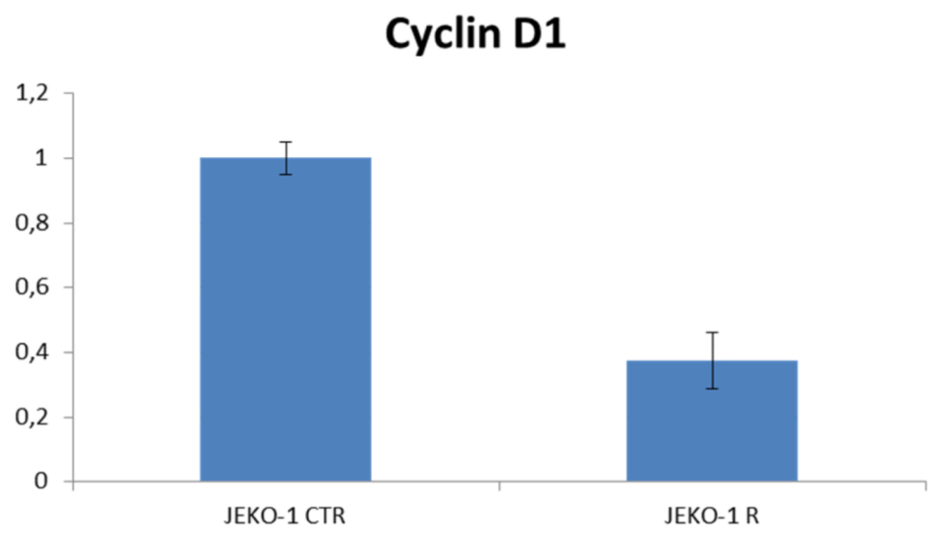

Supplementary Figure 3

Densitometric Analysis of cyclin D1 protein levels in JEKO-1 R cells relative to the parental JEKO-1 cell line (JEKO-1 CTR). Data are normalized for the housekeeping actin and represent the mean ± standard deviation of three independent experiments.

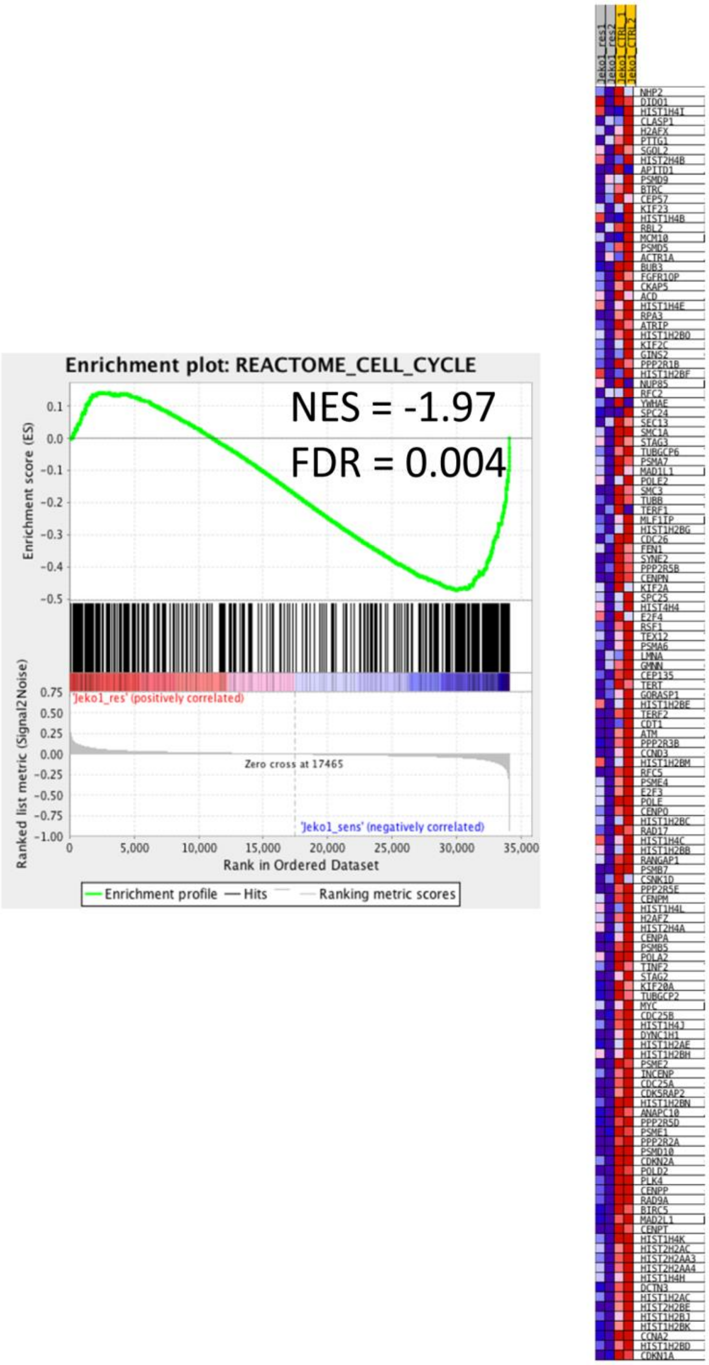

Supplementary figure 4

GSEA plot and the corresponding heatmap of the leading edge genes of a representative gene-sets significantly enriched among the transcripts down-regulated in JEKO1 R cell lines in comparison with parental JEKO1 cell lines. NES, normalized enrichment score; FDR, false discovery rate. The heatmaps show the relative expression values for the genes part of the core enrichment.

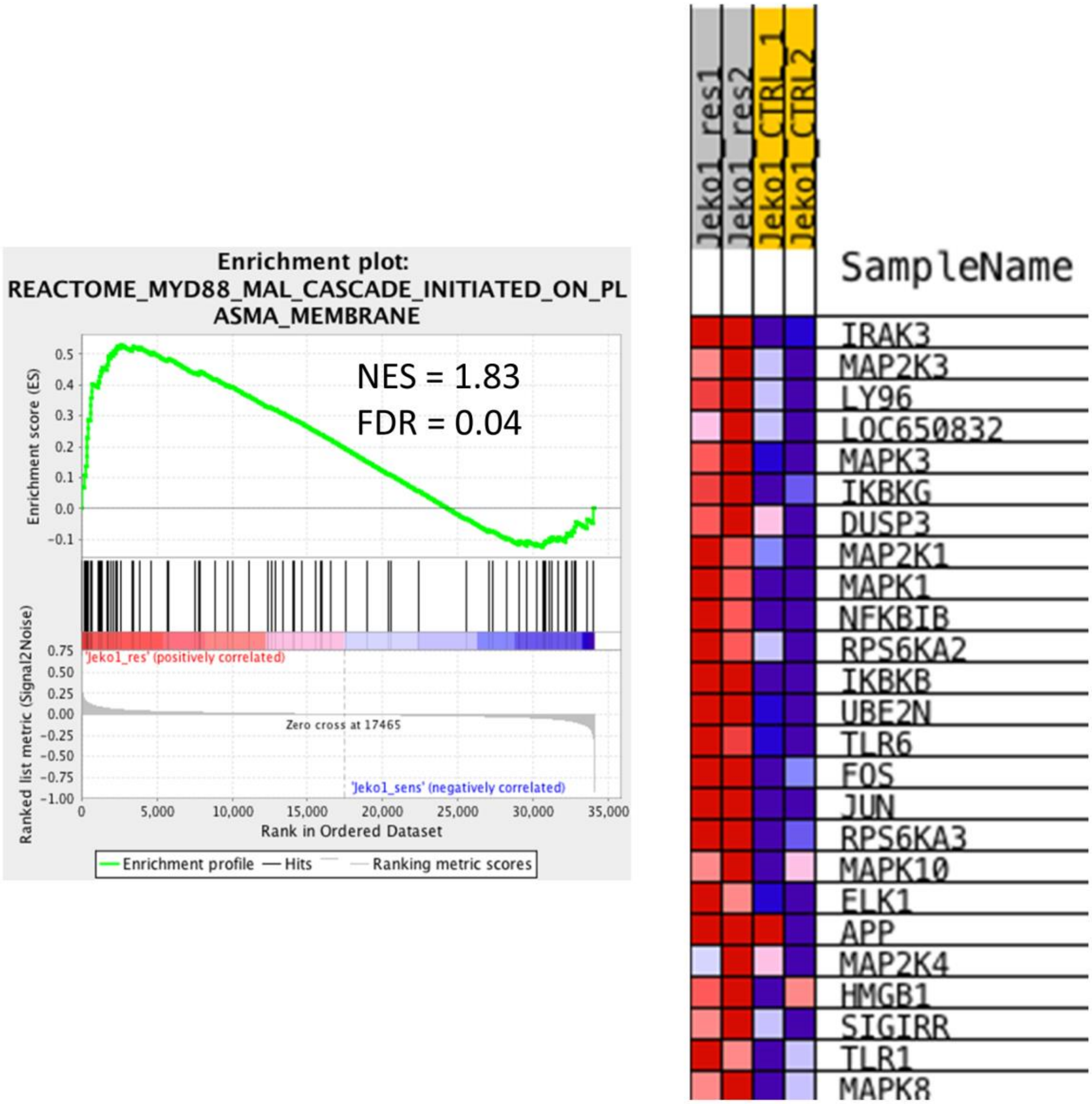

Supplementary figure 5

GSEA plot and the corresponding heatmap of the leading edge genes of a representative gene-sets significantly enriched among the transcripts up-regulated in JEKO1 R cell lines in comparison with parental JEKO1 cell lines. NES, normalized enrichment score; FDR, false discovery rate. The heatmaps show the relative expression values for the genes part of the core enrichment.

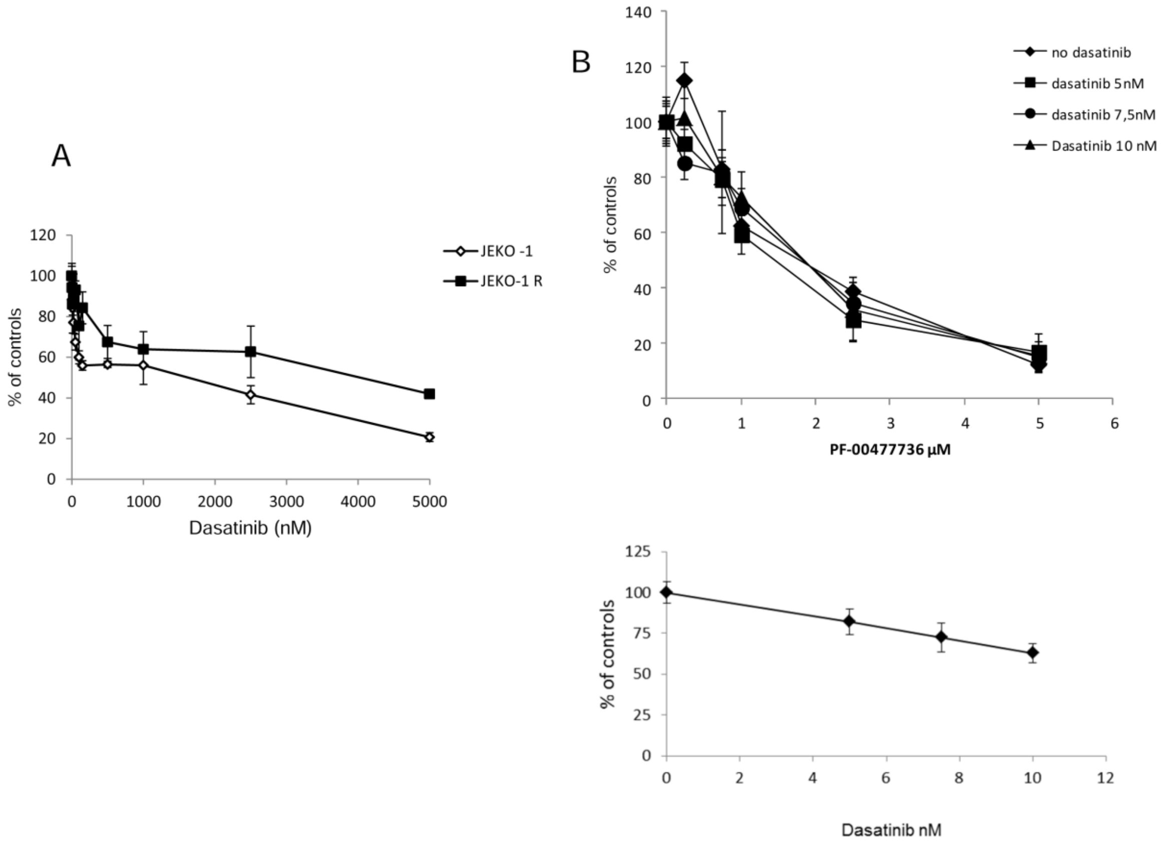

Supplementary figure 6

(A) : Cytotoxic effect of dasatinib in JEKO-1 parental (■) and in JEKO-1 R (▫) cells

(B) : Cytotoxic effect of PF-00477736 in REC-1 cell line either alone or with not toxic concentrations of dasatinib (upper panel). Dose response curve of dasatinib in REC-1 cell line (lower panel).
